# The Eastern Caribbean Health Outcomes Research Network (ECHORN) Cohort Study: Design, Methods, and Baseline Characteristics

**DOI:** 10.3390/ijerph21010017

**Published:** 2023-12-21

**Authors:** Terri-Ann M. Thompson, Mayur M. Desai, Josefa L. Martinez-Brockman, Baylah Tessier-Sherman, Maxine Nunez, O. Peter Adams, Cruz María Nazario, Rohan G. Maharaj, Marcella Nunez-Smith

**Affiliations:** 1 Ibis Reproductive Health, Cambridge, MA 02142, USA; 2 Department of Chronic Disease Epidemiology, Yale School of Public Health, New Haven, CT 06520, USA; mayur.desai@yale.edu; 3 General Internal Medicine, Yale School of Medicine, New Haven, CT 06520, USA; baylah.tessier@yale.edu (B.T.-S.); marcella.nunez-smith@yale.edu (M.N.-S.); 4 School of Nursing, The University of the Virgin Islands, St. Thomas 00802, U.S. Virgin Islands; mnunez@uvi.edu; 5 Faculty of Medical Sciences, The University of the West Indies, Cave Hill, Bridgetown P.O. Box 64, Barbados; peter.adams@cavehill.uwi.edu; 6 Medical Sciences Campus, The University of Puerto Rico, San Juan 00921, Puerto Rico; cruz.nazario@upr.edu; 7 Faculty of Medical Sciences, The University of the West Indies, St. Augustine, St. Augustine, Trinidad and Tobago; rohan.maharaj@sta.uwi.edu

**Keywords:** noncommunicable diseases, chronic disease, United States, Caribbean region, US territories, prevalence, health disparities, biobank, cohort study

## Abstract

Noncommunicable diseases (NCDs) account for a higher proportion of mortality and morbidity in the Caribbean and US territories—majority-minority communities—than in the United States or Canada. Strategies to address this disparity include enhancing data collection efforts among racial/ethnic communities. The ECHORN Cohort Study (ECS), a regional adult cohort study, estimates prevalence and assesses risk factors for NCDs in two United States territories and two Caribbean islands. Here, we describe the cohort study approach, sampling methods, data components, and demographic makeup for wave one participants. We enrolled ECS participants from each participating island using random and probability sampling frames. Data components include a clinical examination, laboratory tests, a brief clinical questionnaire, and a self-administered health survey. A subset of ECS participants provided a blood sample to biobank for future studies. Approximately 2961 participants were enrolled in wave one of the ECS. On average, participants are 57 years of age, and the majority self-identify as female. Data from the ECS allow for comparisons of NCD outcomes among racial/ethnic populations in the US territories and the US and evaluations of the impact of COVID-19 on NCD management and will help highlight opportunities for new research.

## 1. Introduction

Noncommunicable diseases (NCDs) are a leading cause of mortality and morbidity globally [1]. They account for 80.7% of all deaths in the Americas (35 countries, including the United States, Canada, and the Caribbean) [2]. The Caribbean region, which includes more than 25 countries and territories, has the highest NCD burden in the Americas [3]. Common NCDs that contribute to the high burden in the region include cardiovascular diseases, cancer, diabetes, and hypertension [3]. Study findings show a higher prevalence of diseases, such as diabetes and hypertension, in the Caribbean region compared to the United States (US) and Canada [4,5,6] and disparities in the provision of quality care in US territories compared to the US [7].

Many residents and citizens of the United States identify ancestrally and culturally with the US territories and other islands in the Caribbean. According to the 2021 American Community Survey, approximately 13 million US residents reported Caribbean and US territory ancestry [8]. Additionally, historically vulnerable patient populations in the United States share many characteristics with communities in the Caribbean [9,10,11]. Given the shifting demographics in the United States, attention to NCDs in ethnic minority populations can help advance the US agenda to reduce health disparities [12].

Addressing NCDs is a global priority. The Caribbean community’s concerns about the growing problem prompted the first United Nations General Assembly high-level meeting on NCDs [13] in 2011. At this meeting, leaders set goals for new global targets, an action plan for addressing NCDs, and obtaining more regional data to inform efforts to accurately assess the burden of NCDs and develop effective interventions [14]. Meanwhile, US engagement in global NCDs includes agencies such as the Centers for Disease Control and Prevention (CDC) establishing a division dedicated to global NCDs [15,16] and several National Institutes of Health as well as the Millennium Challenge Corporation (a US government corporation) supporting NCD surveillance, research, health projects that address NCDs, training programs, and strengthening country capacity [17,18,19]. Finally, the World Health Organization’s 2013–2020 Global Action Plan for the prevention and control of NCDs set an objective to promote research in low- and middle-income countries [20].

The National Institute on Minority Health and Health Disparities funds the Eastern Caribbean Health Outcomes Research Network (ECHORN), a collaboration among multiple academic institutions and community partners, to increase diversity in NCD research studies as well as bolster capacity for NCD data collection within the Caribbean region. ECHORN is a consortium of four academic institutions: the University of Puerto Rico Medical Sciences Campus in San Juan, the University of the Virgin Islands in St. Thomas and St. Croix, and the University of the West Indies Cave Hill and St. Augustine locations, in Bridgetown Barbados and Port of Spain Trinidad, respectively. The Coordinating Center is located at the Yale University School of Medicine.

ECHORN has a prospective longitudinal cohort study that aims to assess NCDs in four eastern Caribbean islands. The ECHORN Cohort Study (ECS) focuses on risk and protective factors across a wide range of chronic diseases and conditions and compliments cohort efforts on chronic disease in the region, across US territories, and in the United States [21,22,23,24,25]. It is particularly unique because of its broad NCD focus and multi-island partnerships. The data collected from the ECS have broad applicability because these data assess NCDs in a diverse and multi-ethnic community and include populations that account for a significant percentage of the immigrant population in the United States [26]. The ECS collects data, beginning in 2013, from majority racial/ethnic populations, offering an additional comparison group for racial/ethnic minority populations living in the United States and an ability to generalize research findings to diverse populations.

Here, we present a description of the sampling methods, data components, and baseline sample for wave one of the ECS. Data from the ECS may be used to characterize NCD risk and prevalence for cancer, diabetes, and cardiovascular disease for two US territories and two eastern Caribbean islands. Additionally, analyses of the ECS data may contribute to key research agendas, such as patient adherence and self-management, promoting health-related quality of life, and greater information on NCD practice, care, and outcomes [27].

## 2. The ECHORN Cohort Study (ECS)

The ECS, a prospective cohort, comprises Caribbean residents aged 40 years and older from the US territories of Puerto Rico (PR) and the US Virgin Islands (USVI), and independent Caribbean nations, including Barbados (BB) and Trinidad and Tobago (TT). PR is the largest island in the study with a population of 3.2 million, followed by TT (1.5 million), BB (281,835) and the USVI (99,076) [28,29,30,31]. The islands vary in demographics. The official language in BB, TT, and the USVI is English, while PR uses both English and Spanish. In TT and BB, healthcare is free and covered by the government and taxpayers. In contrast, the USVI and PR do not provide universal coverage and have a mix of public and privately financed health insurance plans. Additionally, the gross domestic product per capita in 2019 was $16,000 USD for TT, $18,000 USD for BB, $33,000 USD for PR, and $39,000 USD for the USVI.

The ECS provides (1) an estimated prevalence of diabetes, cancer, and heart disease for the cohort; (2) assesses the effect of known and potential risk factors associated with the development of diabetes, cancer, and heart disease; and (3) explores genetic determinants of NCDs.

## 3. A Collaborative Approach

Establishing a multi-site longitudinal cohort study requires a collaborative approach to protocol development, implementation, data analysis, and dissemination. At the start, we put together research teams in each island, including a principal investigator, faculty fellow, project manager, research assistant, and clinical research nurse. The US-based coordinating center houses staff who provide overall administrative coordination as well as technical expertise. Our research teams and coordinating center work together to acquire ethics approval for the ECS and to design research procedures and instruments. This collaborative approach ensures that the research measures are appropriately adapted to each location and increases the fidelity of research implementation.

We employ a community-engaged research approach throughout the study design, implementation, and translation phases. We work with local researchers and community members to (a) identify and collaborate with local statisticians; (b) source local sampling surveys to create sampling strategies specific to each research site; (c) establish community advisory boards; (d) observe local customs related to research study implementation (such as one site not incorporating participant remuneration); (e) establish research centers in communities that are easily accessible by public transportation; and (f) assess the acceptability of the ECS through the conduct of a pilot study with community members prior to launch.

## 4. Methods

### 4.1. Sampling Strategy

For wave one data collection, we recruit a sample of residents in each of the four island sites. We create sampling frames using national surveys in each island as a basis: The Centers for Disease Control (CDC) Behavioral Risk Factor Surveillance System in the US Virgin Islands; the Barbados Health of the Nation Study and Barbados Labour Surveys; the Central Statistical Office Continuous Household Surveys in Trinidad; and the US Census Track data in Puerto Rico. The ECS sample size is based on the population size of each island, with the intention that larger island sites will contribute a greater proportion of participants to the sample.

Puerto Rico, Barbados, and Trinidad use a stratified multi-stage probability sample design, where residents come from households within randomly selected enumeration districts. In Barbados, we sample residents from 25 randomly selected enumeration districts across the entire island. In Puerto Rico and Trinidad, we sample residents from households within 96 and 70 randomly selected enumeration districts, respectively, in select communities/municipalities in the island. These select communities/municipalities have distributions of age, race/ethnicity, sex, and education level similar to the island’s general population.

For the US Virgin Islands, we use a simple random sampling technique, such that each resident has an equal probability of being selected for the study. In the US Virgin Islands, we invite residents from St. Thomas, St. Croix, St. John, and Water Island to participate in the study by phone using a random digit dialing process.

For all island sites that use a stratified multi-stage probability sampling frame, a single participant is selected from each household. In Barbados, Trinidad, and Puerto Rico, we recruit residents through household visits during the day and evening and on weekday and weekends. In the US Virgin Islands, we recruit residents by phone. We make three attempts to reach an adult at the residence. If there is no response, the number/household is removed from the recruitment list. In the event of multiple eligible residents, we invite the adult with the most recent birthdate. If they decline participation, then we invite the next eligible resident from the household to participate.

The sampling strategy and recruitment protocols for each island site is provided in Appendix B.

### 4.2. Eligibility Criteria

**Figure 1:**
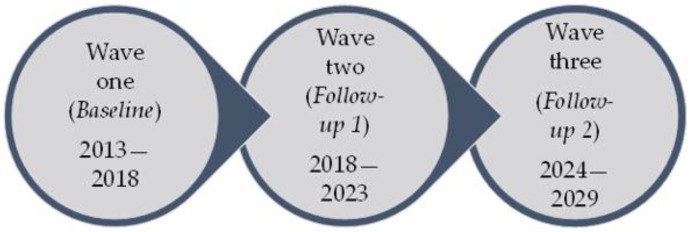
ECS Data Collection Timeline.

We require ECS participants to be 40 years of age or older, live in one of the four islands, speak English or Spanish, and have a reliable contact/residential address and a permanent or semi-permanent resident for at least 10 years with no plans for permanent relocation within five years. Importantly, we do not consider participants’ health status at the time of enrollment when determining eligibility. We verify a resident’s ability to provide informed consent and complete the baseline assessment, by asking them to complete a cognitive test, the mini-cog^4^. We require a score of 1, 2, or 3 with a normal clock drawing test, indicating no cognitive impairment, for enrollment. Once a resident agrees to join the study, we schedule an appointment to visit a community assessment center. Once the baseline assessment is complete, participants in the USVI, TT, and PR are given USD$25 in a gift voucher or cash. Following local guidance, we do not provide incentives to participants from the Barbados site. Wave one participants join the ECS starting in June 2013 and ending in June 2018. Follow-up begins 4–5 years after each participant’s baseline assessment. As participants enter the follow-up window, they are identified and contacted (Figure 1).

#### Data Elements

The ECS harmonizes with other US cohort studies, such as the Jackson Heart Study [21] and Hispanic Community Health Study/Study of Latinos [23], by integrating similar clinical, behavioral, and demographic measures into its design. Data components include a clinical examination, laboratory tests, a brief clinical questionnaire, and a self-administered health survey. The ECS biobank stores genetic information for a subset of participants. The biobank aims to accelerate the identification of new markers of disease and environmental factors associated with NCDs. We invite participants from three of four island sites where biobanking is approved by local institutional review boards to provide a biological specimen for biobanking.

### 4.3. Clinical Exam and Laboratory Tests

We ask wave one ECS participants to complete a clinical examination, which begins with a family history, including a report of NCDs, other medical conditions, and deaths in the extended family. They are also asked to report all medications that they are taking at enrollment and to describe medication adherence. Anthropometric measurements are captured next by trained clinical research nurses. Measures include weight; height; skin fold thickness; waist, hip, and neck circumference; grip strength; resting clinic blood pressure; and an ankle brachial index. All wave one participants provide a blood sample at the beginning of their clinical assessment for immediate testing. Blood tests for lipids (fasting or non-fasting), a complete blood count, hemoglobin A1C, and a comprehensive metabolic panel are performed. See Table 1 for details. Given the geographical distance between island sites, no central laboratory is used to process blood samples. We use point of care machines for blood sample processing at 3 of our 4 island sites. In Puerto Rico, we use a clinical laboratory at the Hispanic Alliance for Clinical & Translational Research in Puerto Rico (formerly the Puerto Rico Clinical and Translational Research Center). Participants can share the results of laboratory tests with their primary care providers.

### 4.4. Self-Reported Health Survey

We also ask wave one ECS participants to complete a health survey in English or Spanish using a keyboard or touch screen. We use the Questionnaire Development System (QDS) software (Nova Research, version 2.6.1.2) to collect baseline survey data. Audio computer assisted self-interviewing (ACASI) is made available to those participants who want the survey read aloud to them. For those participants who are not comfortable with the computer-assisted interview, we allow a member of the research team to read the survey aloud to them. In addition to demographic variables, such as age, sex, marital status, employment, and sexual orientation, we collect data on several factors potentially associated with NCDs. Alcohol use, smoking, physical activity, sleep habits, reproductive history, access to care, chronic disease history, depressive symptoms, and migration history are some of the factors collected. A modified version of the National Health and Nutrition Examination Survey (NHANES) Dietary Screening Questionnaire (DSQ) [32] is used to assess nutritional intake. Finally, we ask all participants to provide information on the most influential members of their health network as it relates to health information, advice, and health decisions. Participants vary in terms of survey completion, with most taking between 60 and 180 min. Table 1 outlines the baseline data by collection method.

## 5. Data Quality Assurance Procedures

To ensure consistency in data collection across our island sites, the medical director at the coordinating center trains all clinical research nurses on the clinical procedures at an in-person meeting (June 2012) and offers refreshers through online videos and training sessions. Further, a manual of operations, which offers visual depictions, detailed descriptions of each clinical measure, and instructions for each procedure along with special considerations and potential modifiers to facilitate proper measurement, is shared with the clinical research staff. All other research staff receive training from the coordinating center on the research protocols (between June 2012 and May 2013). Additionally, each island site pilots the ECS baseline protocol, from intake (inclusive of laboratory testing) to exit with 3–4 community members. This ensures that research staff are confident in the process at launch, but also provides an opportunity to resolve issues with the research protocol. In Puerto Rico, only certified clinical research nurses employed by the Alliance collect the clinical observations (see Table 1) and complete the blood draw. At all other research sites, clinical research nurses are part of the research team, performing the clinical examinations and phlebotomy. Laboratory data are entered into a REDCap database. Data from all sources are doubly entered, cleaned, and merged into SAS (SAS Institute, Carey, NC, USA). Data quality assurance, storage, cleaning, and analysis are conducted at the coordinating center. Greater details on the clinical data in the ECS can be found in Appendix A.

Requests for data and data analysis are managed by the Data Access and Scientific Review (DASR) committee. Access to the manual of procedures is contingent on approval from the DASR committee. The DASR committee is made up of the five principal investigators, the ECHORN Coordinating Center Director (Chair; non-voting member), and ad-hoc members with expertise in epidemiologic study design and biostatistics (non-voting members).

## 6. Results

### 6.1. Description of the ECS Wave One Sample

In total, wave one of the ECS has 2961 participants, reflecting an overall participation rate of 70%. Participation at all island sites is above 50% (Table 2).

The majority of wave one participants identify as female and are on average 57 years old (range 40–91). Close to 62% of the sample rate their general health as ‘good’ or ‘very good’. Those who did not enroll in the cohort were slightly younger (56 vs. 57 years, *p* < 0.05) and more likely to be male (39% vs. 35%, *p* < 0.05). See Table 3 for demographic and health characteristics of ECS wave one participants.

### 6.2. The Biobank

Three of the four island sites are a part of the biobank: PR, BB, and the USVI. We divide our blood samples into fifteen 0.5 mL aliquots, store them temporarily at −80 °C on-site, and then ship them to the coordinating center for permanent storage. All aliquots are stored at the coordinating center because of concerns power and back-up power to the −80 °C freezers in the island sites. Biobank samples are stored for a short time in-island and then sent, using dry ice, to the US-based coordinating center for long-term storage. We divide the biobank samples into multiple aliquots to ensure many investigators can use the samples to examine environmental chemicals and nutritional biomarkers and draw causal links to disease.

A total of 635 participants are included in the biobank: 10% of participants in BB (n = 101), 27% in the USVI (n = 95), and 57% in PR (n = 439). Nearly two-thirds (65%) of the biobank participants are female, and approximately 64% of biobank samples are from participants ages 50–69 years. About 15% of the samples are from participants 70 years and older. The biobank complements efforts already underway in the United States, such as the National Biomonitoring Project at the CDC, to better understand factors associated with NCD development. The number of samples by type and island site are shown in Table 4.

## 7. Discussion

The Caribbean and US territories in the Caribbean Sea as a region is home to diverse multiethnic communities that bear many similarities with populations in the US. However, a paucity of health outcomes research on Caribbean and Caribbean diasporic populations limits our understanding of the many disparities that affect these populations. The ECS, a research initiative from a NIMHD-funded consortium, bolsters efforts to conduct NCD research with racial/ethnic minorities and strengthens workforce capacity to monitor NCD prevalence and risk. The ECS is particularly relevant given increased attention to NCD prevention and management following the COVID-19 pandemic [2]. The ECS will offer data on the impact of the pandemic on NCDs in this population, help identify points for intervention, and highlight opportunities for new research.

The ECS complements ongoing cohort research in the United States by incorporating similar clinical, behavioral, and demographic measures. Further, it offers the opportunity to compare health outcomes across multiple groups within the cohort and across cohorts. Using data from the ECS, researchers will be able to compare health outcomes for the US territories versus non-US Caribbean sites, compare the ECS to extant datasets focused on racial/ethnic populations in the US, and compare the ECS with emerging datasets and projects.

In addition to providing information on the minimum NCD indicators (nutrition, alcohol and tobacco use, physical activity, blood pressure, weight, cholesterol, and healthcare seeking), the ECS collects information on novel risk/protective factors, such as sleep and use of alternative medicine, and can assess the role of health networks on health behaviors. Further, the rich diversity of the ECS sample allows researchers to stratify risks by several key social characteristics, such as income, gender, and racial/ethnic self-identification. Finally, the ECS biobank facilitates the identification of new biomarkers for chronic diseases.

Another strength of the ECS lies in its application of a regional approach and its use of a community-engaged approach in the development and implementation of the study. A common challenge to NCD research efforts in the Caribbean region is that countries with limited resources have difficulty achieving economies of scale. Regional coordination allows for the collection of data on multiple NCDs at multiple sites. A second advantage of the regional approach is the ability to assess and highlight similarities and differences in NCD risk based on contextual and clinical factors across the eastern Caribbean. Finally, the regional approach facilitates health information exchange and the integration of interventions across multiple countries.

Community-engaged methods center the community’s assets and strengths and ensure that programs, policies, and studies that emerge are sustainable, effective, and relevant. We used site-specific statisticians and research teams as well as community advisory boards to promote the use of local knowledge and expertise for our sampling strategies, study instruments, and study implementation. Furthermore, the use of localized research teams to collect data and a standardized research protocol ensures the longevity of the study by supporting ancillary studies on NCDs in the same islands, expansion to other islands in the region, and research initiatives that respond to changes in the pattern of the NCD epidemic. As an illustration, in response to reports of increasing numbers of children being diagnosed with chronic diseases, such as diabetes and hypertension, ECHORN is now recruiting the children and grandchildren (ages 5–17 years) of existing ECS participants, establishing the first multi-island intergenerational cohort study in the region.

There are notable limitations to the ECS. Importantly, the sample size of the ECS does not allow us to generate prevalence estimates for each island in the study. Further, some clinical data elements may be missing for a portion of participants. Budgetary limitations led some island sites to shift from using reference laboratories to point of care machines. During that shift, blood sample collection was halted at those sites. Biobank samples are only available for three of the four island sites. Trinidad did not receive approval from the local institutional review board to contribute biospecimens to the biobank. Additionally, St. Croix did not have a −80 °C freezer on site to store samples, so biospecimen collection in the USVI is limited to St. Thomas. Finally, the effectiveness of recruitment efforts varied by island site as shown in Table 2.

## 8. Conclusions

The ECS, a regional cohort study, evaluates the prevalence of and risk factors for heart disease, diabetes, and cancer among adults residing in two US territories and two Caribbean islands. Clinical, behavioral, and demographic data are available for approximately 2961 participants. Further, the ECS stores biological specimens for 635 participants, which may aid in the identification and management of noncommunicable diseases. Data from the ECS are an important contribution to the United States’ efforts to reduce health disparities because it offers insights into biological, clinical, and behavioral factors associated with NCD health outcomes among racial/ethnic minorities. Considering the growing proportions of the US population reporting Caribbean lineage, analyses of ECS data will shed light on similarities between US residents and those in the US territories and signal contextual differences that may place residents in the US at greater risk for NCDs. The ECS is the flagship cohort study of ECHORN. ECHORN welcomes requests for ancillary studies, data analyses, and scientific collaborations using the ECS data and research infrastructure. The second wave of data collection (first wave of follow-up) is nearly complete, and we are actively planning for wave three.

## Figures and Tables

**Table 1:** Components of ECS wave one data collection.

Components	Clinical Interview	Clinical Exam	Questionnaire
**Clinical Observations**			
Medical History	X		
Social Network	X		
Medication Use & Adherence	X		
Height and weight		X	
Waist, hip, and neck circumference		X	
Grip strength		X	
Resting blood pressure		X	
Ankle brachial index		X	
*Laboratory tests*			
Lipid panel		X	
Complete blood count		X	
Hemoglobin A1C		X	
Metabolic panel		X	
**Survey Elements**			
Demographics (e.g., age, sex, gender identity, marital status, parity, etc.)			X
Global Health (general health assessment)			X
*Physical and Mental Health*			
History of chronic diseases			X
Health care access and utilization			X
Reproductive health			X
Depression			X
*Health behaviors*			
Substance use			X
Tobacco use			X
Physical activity			X
Nutritional assessment			X
Sleep			X
*Social Health*			
Incarceration			X
Social support			X
Neighborhood factors			X

**Table 2:** ECS wave one participants enrolled by island site and recruitment method.

Recruitment Method	Site	Encountered	Eligible	Enrolled	Participation Rate
Household	BB	2317	1415	1008	71%
Household	PR	1192	988	771	78%
Household	TT	1326	1235	829	67%
Random Digit Dialing	USVI	1022	618	353	57%
	Total	5857	4256	2961	70%

**Table 3:** ECS wave one participant characteristics (N = 2961).

Characteristic	N (Available Data)	Mean (SD)
*Age* (years)	2961	57.3 (10.3)
*BMI* (Kg/m^2^)	2923	29.2 (6.1)
*Total cholesterol* (mmol/L)	2372	193 (39.3)
*HDL* (mmol/L)	2345	52.6 (14.6)
		**N (%)**
*Sex*, Female	2961	1935 (65.4)
*Age* (years)	2961	
40–49		742 (25.1)
50–59		1046 (35.3)
60–69		775 (26.2)
70+		398 (13.4)
*Marital status*	2901	
Single		1181 (40.7)
Married		1196 (41.2)
Divorced or separated		304 (10.5)
Widowed		220 (7.6)
*Sexual orientation*	2792	
Heterosexual (‘straight’)		2488 (89.1)
Gay or lesbian		25 (0.9)
Bi-sexual		17 (0.6)
Not sure or questioning		262 (9.4)
Regular source of care (*Is there one place you usually go when you need routine or non-emergency care?*)	2934	
Yes		2093 (71.3)
I do not seek routine care anywhere		405 (13.8)
I seek routine care at more than one place		436 (14.9)
General Health (*In general, would you say your health is:*)	2956	
Poor		84 (2.8)
Fair		800 (27.1)
Good		1267 (42.9)
Very Good		545 (18.4)
Excellent		260 (8.8)
*Comorbidities*		
Obese (BMI 30+)	2923	1136 (38.9)
Hypertension ^a^	2953	1459 (49.4)
Cardiovascular Disease ^b^	2956	410 (13.9)
Diabetes ^c^	2503	562 (22.5)
Cancer	2953	121 (4.1)
Current Smoker	2843	230 (8.1)
*At least one of the comorbidities above*	2829	2213 (78.2)
*PAD* (ABI ≤ 0.9)	2772	121 (4.4)

^a^ Hypertension is self-reported. Baseline rate also includes borderline or pre-hypertension. ^b^ Cardiovascular disease is self-reported and includes coronary heart disease, angina, abnormal heart rhythm, heart attack, stroke, or congestive heart failure. ^c^ Diabetes is self-reported.

**Table 4:** The ECS biorepository by type of sample and island site.

	All Sites		BB		PR		USVI	
Biobank participants	635		101		439		95	
Sample type	n	%	n	%	n	%	n	%
Blood clot	633	5.93	104	5.99	435	5.87	94	6.13
Serum	3098	29.03	472	27.20	2201	29.72	425	27.72
Whole blood	6942	65.04	1159	66.80	4769	64.40	1014	66.14

## Data Availability

The data presented in this paper are available on request from the corresponding author. Requests for ECS data and/or analyses of ECS data may be requested through the Data Access and Scientific Review (DASR) committee (https://www.echorn.org/request-echorn-data).

## Appendix A. Detailed Descriptions of Clinical Observations Included in Wave One of the ECHORN Cohort Study (ECS)

**Table A1:** ECS wave one clinical observations: measures, data available, and definition.

Clinical Observation	Equipment Used	Measure	Data Available	Definition
Medical history	Paper forms (later uploaded to a database)	-	2961	A record of the diseases, health conditions, and deaths in the participant’s nuclear and extended family
Medication use and adherence	Paper forms (later uploaded to a database)	-	2961	Medication use: A record of the medications the participant is currently taking (prescribed and non-prescribed), reason for use, dosage, timing, and frequency Medication adherence: the extent to which the participant conforms to the provider recommendation for a prescription (timing, dosage, and frequency)
Weight	Tanita BF-689 digital scale	Kilometers	2923	A measure of relative mass
Height	Seca 213 mobile Stadiometer	Centimeters	2923	A measure of how tall the participant is, taken from a standing position
Waist, hip, and neck circumference	Seca 201 tape measure	Centimeters	2936, 2935, and 1723, *respectively*	A measure of visceral fat
Grip strength	Jama hand dynamometer	Kilograms	2930	A measure of the maximum isometric strength of the hand and forearm muscles
Resting blood pressure	Watch BP office ABI device Blood pressure cuffs (various sizes)	Millimeters of mercury	2932	A measure of the force of blood on the artery walls
Ankle brachial index	Watch BP office ABI device Blood pressure cuffs for upper arm and ankle (various sizes)	Ratio	2898	A measure of the ankle (posterior tibial artery) systolic pressure divided by the brachial artery systolic pressure
Lipid panel	Point of care machine or reference laboratory	-	2345	Measures of cholesterol levels and a measure of triglycerides *Participants were not required to fast prior to participating*
Complete blood count	Point of care machine or reference laboratory	-	1434	Measures of red and white cells, hemoglobin, hematocrit, and platelets in the blood
Hemoglobin A1C	Point of care machine or reference laboratory	-	2350	Measure of blood sugar levels *Participants were not required to fast prior to participating*
Metabolic panel	Point of care machine or reference laboratory	-	1890	A measure of several substances in the blood, including glucose, calcium, blood urea nitrogen, creatinine, sodium, potassium, and albumin

## Appendix B. Detailed Descriptions of the Sampling Methodology for the Island Sites Included in Wave One of the ECHORN Cohort Study (ECS)

### Appendix B.1. Barbados

#### Appendix B.1.1. Sampling Frame and Methodology: Barbados

ECS recruitment in Barbados used a stratified two-stage design with probability proportional to size (PPS) sampling of EDs (the primary sampling units or PSUs) in the first stage and recruitment of households with systematic sampling after a random start in the second stage. A single eligible participant is selected from each household.

#### Appendix B.1.2. Sample Size/Sampling Fraction

ECHORN Barbados aims to recruit 1008 participants aged 40 and older. Using the Barbados Statistical Service estimate of the 2010 Barbados population [33], there were 277,821 people in Barbados; 127,798 (43.9%) of whom were 40 years or older.

#### Appendix B.1.3. Enumeration Districts (Primary Sampling Units)

The Barbados Statistical Service (BSS) has organized Barbados into 583 enumeration districts (EDs) with an average of approximately 180 households per ED. Previous surveys (notably the rolling Barbados Labour Force Survey and the ongoing 2012 Barbados Behavioral Risk Factor Survey—The Health of the Nation) have each selected 45 EDs for survey recruitment but require greater numbers of participants than the ECS. Using an earlier survey (The Survey on Health, Well-Being and Aging in Latin America and the Caribbean), estimated ED design effects for key demographic and self-reported health outcome variables range from 0.95 to 1.2, reflecting the relative within-ED heterogeneity in Barbados [34].

#### Appendix B.1.4. Sample Stratification

Sample stratification is based on four geographical island subdivisions used by the BSS, with EDs within each subdivision “reasonably homogenous in terms of social and economic development”. The geographical strata are provided in the Table below.

**Strata**	**Stratum One**	**Stratum Two**	**Stratum Three**	**Stratum Four**	**Total**
Parishes in Stratum	St. Michael	Christ Church St. Philip	St. James St. George St. Thomas	St. Lucy St. Peter St. Andrew St. Joseph St. John	
Total possible EDs	181 (31%)	176 (30%)	130 (22%)	96 (17%)	583 *
Number of Sampled EDs (45 EDs)	14	14	10	8	45
Number of Sampled EDs (35 EDs)	11	11	8	6	35
Number of Sampled EDs (25 EDs)	8	8	6	4	25
* The total will be 538 EDs (583–45 being used by the Barbados Statistical service and the Health of the Nation Study).					

#### Appendix B.1.5. Non-Response Rate

Few population-based studies and only one cohort study has been performed in Barbados. The Barbados Eye Studies (BES) is based on a nationally representative simple random sample of 4709 Barbados-born citizens aged 40 to 84 years at baseline (84% participation) [35]. Surviving cohort members are reexamined 4 and 9 years later, with 85% and 81% participation, respectively. Therefore, a 20–30% non-response and dropout estimate was anticipated.

#### Appendix B.1.6. Sampling Design

STEP 1: Twenty-five EDs were chosen from the 538 EDs in the sampling frame using probability proportionate to size (PPS) methodology. This means the chance of an ED being chosen will be in proportion to its 40 years of age and over population size.

STEP 2: Maps of selected Eds were provided by the Barbados Statistical Services (BSS), and the number of households selected from each ED will be in proportion to population size. Households were systematically sampled at an interval of approximately 1 in 4. If the recruitment quota was not met for a specific ED, research members resampled the ED starting from a different number while observing the 1 in 4 interval.

STEP 3: A single eligible participant was selected from each household using the ECS eligibility criteria: (1) ≥40 years of age at the time of recruitment, (2) resident in the island for ≥10 years, (3) have no plans to permanently relocate from the island for ≥5 years, (4) able to provide informed consent, (5) English or Spanish language speaking, and (6) have stable contact residential information.

The selection of the household member was performed as follows: (1) The sex, age and date of birth of each household member 40 years of age and over were recorded on a recruitment form. (2) The person who last celebrated a birthday was selected. In the case of twins, the older sibling was selected. In the case of people with the same birthday, the older of the two was selected.

In households with more than one person meeting the eligibility criteria above, should the first selected person decline participation, an attempt was then made to enroll a second eligible person within the household. The person with the most recently celebrated birthday was approached. If this person also decline participation, no further attempts were made to enroll persons from that household.

In cases where (1) an eligible person was not willing to participate in the study, (2) there was no eligible person at the house or (3) the property was a business or unoccupied house, the recruiter moved on to the property at the next interval.

If the property was an apartment block or a multifamily unit, the strategy used by the Barbados Statistical Service was employed. Otherwise, for apartment blocks, we made a random start on the top floor of the apartment complex and then every unit at the chosen sampling interval was approached.

Homes were visited up to 3 times if necessary. If no one was at home at the time of the first visit, 2 subsequent visits at different times/days, including, if possible, an evening and a weekend, were made. If no adult was reached after three attempts, the household was removed from the list.

Once an eligible person was selected, contact information (name, address, and phone numbers) was collected. An appointment was set up along with a follow-up reminder phone call.

### Appendix B.2. US Virgin Islands

#### Appendix B.2.1. Sampling Frame and Methodology: US Virgin Islands

ECS recruitment in the US Virgin Islands used a simple random sampling technique to recruit participants from St. Thomas, St. Croix, St. John, and Water Island. The population base of the US Virgin Islands is relatively small and scattered. Additionally, geographic markers and community boundaries are not uniformly nor officially demarcated, and the variables of interest in this study are not restricted to community dwellers on any one island or section of that island. Therefore, a whole island (all 4 islands) recruitment approach was recommended.

#### Appendix B.2.2. Sample Size

ECHORN US Virgin Islands recruited 353 participants aged 40 years and older. The 2010 US Census Bureau estimated the population to be 106,405 with 51,634 on St. Thomas, 50,601 on St. Croix, 4170 on St. John, and 182 on Water Island. Approximately, 43,505 (41%) were between 40 and 69 years of age [36].

#### Appendix B.2.3. Sampling Design

STEP1: Random digit dialing of a bank of telephone numbers for landline and mobile telephones was performed. This sampling methodology approximates that used when conducting the Behavioral Risk Factor Surveillance Survey (BRFSS) in the US Virgin Islands [37]. This methodology allowed access to the broadest base of US Virgin Island residents.

The area code for all numbers in the USVI is 340. To the best of our determination, the following numbers represented prefixes for mobile and landlines on St. Thomas and St. John: 201, 227, 228, 244, 344, 422, 473, 474, 513, 514, 626, 642, 643, 677, 690, 693, 714, 715, 727, 770, 774, 775, 776, 777, and 779. The following numbers represented prefixes for mobile and landlines on St. Croix: 220, 226, 277, 332, 692, 712, 713, 718, 719, 771, 772, 773, and 778.

STEP 2: Using a table of random numbers, the last four digits (suffix) of a telephone number were selected. Prefixes were sorted according to island/district. The selection of a prefix for each telephone number was determined using random sampling with replacement. The prefixes were then sequentially and systematically matched with the telephone number’s last four digits, which were previously selected from a table of random numbers.

STEP 3: The selection of a household member was performed as follows: (1) Research staff contacts the household. (2) When a person was reached, a prepared script was read primarily to inform the person about the study and the inclusion requirements for study eligibility. Households with persons who met the ECS criteria, ≥40 years of age at the time of recruitment, resident in the island for ≥10 years, have no plans to permanently relocate from the island for ≥5 years, able to provide informed consent, English or Spanish-language speaking, and have stable contact residential information, could participate in the study. At the time of recruitment, individuals were provided with an appointment at a Community Assessment Center.

If more than one person in the household was 40 years or older, then the person who most recently celebrated a birthday was approached. In the case of twins, the older twin was selected; if two people have the same birthday, then the older of the two was selected. In households with multiple eligible persons, if the originally selected person declined participating in the study, attempts were made to enroll a second eligible person within the household.

If a contact person is not reached when a call is placed, two additional attempts at a different time and day were made. If after three attempts, a contact person was not reached, the number was retired from the list of randomly selected telephone numbers.

If the number dialed reached any location other than a private residence or mobile number, the call was abandoned and recorded onto a tracking form. In the event that a landline was called, a minor answered the telephone and an adult was not at home at the time, research staff called back another time to try and reach an adult. If no adult was reached after three attempts, the number was retired. If a minor’s mobile number was reached, the number was abandoned and recorded onto a tracking form.

The protocol for when a voicemail was reached instead of a person was as follows. If a person does not answer the telephone, we disconnected the call. The telephone number was called a total of three different times of the day, but no voice messages were left.

### Appendix B.3. Puerto Rico

#### Appendix B.3.1. Sampling Frame and Methodology: Puerto Rico

ECS recruitment in Puerto Rico used a stratified multi-stage design. Three barrios within a single municipality were selected. A systematic sampling design was employed within blocks per barrio and a random selection of segments (12 households). A single eligible participant was selected from each household.

#### Appendix B.3.2. Sample Size

ECHORN Puerto Rico recruited 771 participants aged 40 years and older. According to estimates of the US Census Bureau, 2006–2010 American Fact Finder [38], the Archipelago of Puerto Rico had a population of 3,762,322 inhabitants, and San Juan Municipality had 404,748 inhabitants. A similar percentage distribution by sex was observed: 46% and 48% were males in San Juan and Puerto Rico, respectively. The median age (years) reflects slightly older dwellers in San Juan (38.2 years) than in Puerto Rico (35.9 years).

#### Appendix B.3.3. Enumeration Districts (Primary Sampling Units)

Selected municipality/community: The municipality of San Juan was selected as the target municipality in Puerto Rico since the inclusion of residents outside this metropolitan area imposed great risk in terms of refusal rates and increased costs within finite fiscal and human resources. San Juan has 18 barrios: Caimito, Cupey, El Cinco, Gobernador Piñero, Hato Rey Central, Hato Rey Norte, Hato Rey Sur, Monacillo, Monacillo Urbano, Oriente, Barrio-Pueblo, Quebrada Arenas, Sabana Llanas Norte, Sabana Llanas Sur, San Juan Antiguo, Santurce, Tortugo, and Universidad. There are 161 Census Tracts listed for San Juan Municipality by the American Fact Finder, US Census Bureau [38].

The primary legal divisions of Puerto Rico are termed “municipios”. For data presentation purposes, the Census Bureau treats a “municipio” as the equivalent of a county in the United States. But in Puerto Rico, a “municipio” behaves administratively like that of a city in the United States, with its own Mayor and Municipal legislature. The Census Bureau recognizes “barrios” as the primary legal divisions of “municipios”. These entities are similar to the minor civil divisions (MCDs) used for reporting data in 29 states of the United States.

There are 1,227,039 households in Puerto Rico and 150,331 households in the municipality of San Juan, but the average family size is practically similar (3.6 and 3.3, respectively). Some economic indices vary when San Juan municipality is compared to Puerto Rico: a higher percentage of the population of San Juan has attained at least a high school diploma, (73.7% vs. 67.6%), a lower percentage of the population of San Juan was classified below the poverty level (39.1% vs. 45.0%), and a higher median income (US dollars) is noted for the population of San Juan compared to Puerto Rico ($14,366 vs. $11,931). In terms of educational attainment, Monacillo has the highest percentage of the population with at least a high school diploma (90.4%), while San Juan Pueblo has the lowest (53.1%). Barrio El Cinco has the lowest percentage of the population under the poverty level (16.9%), whereas barrio Oriente has the highest (54.9%). The San Juan barrio with the highest median income (US dollars) was listed as Caimito ($25,114), and the lowest is barrio Oriente ($9947).

San Juan barrios Santurce, Caimito and Cupey were selected considering a combination of strategies that include the deliberate selection of community areas (in discussion with consulted experts) to increase efficiency and the random selection of households within those areas to reduce selection bias and improve precision of estimated parameters. The selection was based on the expected variability of risk factors and the incidence of NCDs across these geographical areas and barrios, as they could be inferred by their socio-demographic characteristics.

There are 34 census tracts (as defined by US Census 2010) and 1129 blocks (315 blocks without population) in Santurce. Caimito barrio has 20,906 inhabitants and 9234 households, while Cupey has 36,650 inhabitants living in 15,690 households. Both barrios have an average family size of 3.4 but a higher percentage of the population with at least a high school diploma and higher median income when compared to San Juan municipality. There are 6 census tracts and 292 blocks (58 blocks without population) in Cupey and 8 census tracts and 358 blocks (69 blocks without population) in Caimito.

#### Appendix B.3.4. Non-Response Rate

Based on the experience in Puerto Rico with different population studies, we assumed a 25% rejection rate and 10% illegibility rate.

#### Appendix B.3.5. Sampling Design

STEP 1: The sampling frame was defined by the census blocks (1779 census blocks) within census tracts (48 census tracts) in the three barrios of San Juan. Within each barrio, 83 block segments were randomly selected.

STEP 2: All census blocks were listed, and the number of occupied households were identified. The census blocks were ordered and listed by the block’s socioeconomic level (indexed by educational attainment level as the percent of population with at least a high school diploma, and average income in the census tract). There were 1124 blocks in Santurce, 358 blocks in Caimito, and 292 blocks in Cupey.

After blocks were ordered and listed by educational attainment (block average), blocks were systematically selected as follows: 1 out of every 37 blocks from Barrio Santurce, for a total 30 blocks from this barrio; 1 out of every 8 blocks from Barrio Caimito, for a total of 45 blocks from this barrio; and 1 out of every 14 blocks of Barrio Cupey, for a total of 21 blocks from this barrio.

Field workers visited selected blocks to enumerate all households within the selected block. The address (street, house number, etc.) was included in the enumeration list along with any other description that could improve the identification of the dwelling. The block boundary was defined, and commercial buildings and other buildings that are not permanent living dwellings or unoccupied were identified in the segment. Segments of 12 households (HHs) were delineated and identified in each block. In case a census block had less than twelve households, the block was merged with the bordering or adjacent block.

Segments of 12 HHs were randomly selected from selected blocks using a simple random sampling process. In each barrio, 83 segments were randomly selected, and all 12 households within the selected segments were visited. Following this process, consecutive households in each randomly selected segment of the census block were identified by a code number.

STEP 3: The research staff visited the randomly selected segments. We identified the first household (HH#1) in the field by the address in the enumeration list of the selected segment and block number. If HH#1 included more than one dwelling (i.e., a structure with 3 floors that holds different families or residents), based on the number visible electricity meters (AEE), we identified the uppermost residence (in this example, the residence in the third floor) as household number 1 (HH#1) as the initial point of reference. For the segmentation process, each family of each floor was considered as one household. If the building was a condominium with more than one apartment per floor, we identified the apartment located to the extreme right of the highest floor as HH#1 and thus the initial point of reference; for the segmentation process each doorbell was considered as one household.

If the initial point of reference was an eligible dwelling, we tried to establish initial contact with a resident in the household. If there was an adult (>18 years) present in the household, we documented the composition of the residents to identify the number of potential participants that could comply with inclusion criteria for the ECS. If more than one person in the household was 40 years or older, then the person who last celebrated a birthday was approached. In the case of twins, or two individuals with the same date of birth, the older twin or the older person was selected.

If there was no potentially eligible participants in the household, we moved to the next house to the left (facing the door of the last house visited in the block). This process was repeated until the quota of twelve households per block was accomplished.

If no adult was present during the first visit, we documented the result of the visit (date and time of the visit and that no one was present) and left a letter. We then walked to the left (facing the door of the last household visited) towards the next household or apartment. If there was more than one floor, we repeated the process from the highest to the lowest floor.

In those residences where no one opened the door or where a letter was left, we visited on a different day of the week and time than the previous visit. Up to three (3) visits were made to the household. At each visit, we documented the result of the visit. If no adult was reached after three attempts, the household was removed from the list.

### Appendix B.4. Trinidad

#### Appendix B.4.1. Sampling Frame and Methodology: Trinidad

ECS recruitment in Trinidad used a stratified multi-stage design. A random sample of enumeration districts (EDs) from a specified geographic area was created in the first stage, and households were recruited using systematic sampling after a random start in the second stage. A single eligible participant was selected from each household.

#### Appendix B.4.2. Sample Size/Sampling Fraction

ECHORN Trinidad and Tobago recruited 829 participants aged 40 years and older. Population estimates in 2011 for Trinidad and Tobago placed the population at 1,328,019. Approximately 520,321 individuals, close to 50% of the population, are over the age of 40 years [39].

#### Appendix B.4.3. Enumeration Districts (Primary Sampling Units)

Selected municipalities/communities: The geographic area selected in Trinidad for the ECS had the following boundaries: West, the district of Maracas/St. Joseph; East, the district of Caura; North, foothills of the Northern Range; South, the Caroni Savannah Road and surrounding communities. Villages and towns included in this area are Curepe, St. Augustine, Tunapuna, Pasea, Caura, and St. Helena.

The geographic area selected captured the following:

1. The ethnic diversity of Trinidad with a mix of urban and semi-rural; upper-, mid-, and low-income households; and East-Indian, African, and Mixed-race heritage
2. A densely populous area with over 31,000 households
3. Approximately, 155 enumeration districts (EDs), and each ED has between 150 and 300 plus households
4. A well-developed infrastructure of roads, public transportation, water supply, and telecommunication
5. Mature communities with relatively little new construction.

#### Appendix B.4.4. Non-Response Rate

We anticipated a 40% nonresponse rate based on the Tobago Health Study [40].

#### Appendix B.4.5. Sampling Design

STEP 1: A random sample of enumeration districts (EDs) from the geographic area demarcated above was created using the total list of EDs obtained from the Central Statistical Office (CSO).

STEP 2: Maps of the selected EDs were purchased from the CSO, and a starting point was chosen as the first household to be approached. These starting points were already displayed on the maps provided by the CSO. Once the random starting point was chosen, a fixed multiple of every 4th-6th household was approached to obtain a maximum of 40–50 households per ED. For EDs with more than 300 HHs, every 6th HH was chosen; for EDs with between 200 and 300 HHs, every 5th was chosen. For EDs with 200 or less HHs, every 4th was chosen. This approach followed from a discussion with the Central Statistical Office in Trinidad on the Continuous Sample Survey of Population (CSSP) and a review of completed national surveys including the STEPS survey [41,42].

From the initially selected 155 EDs, a random sample of 70 EDs was created.

STEP 3: A single eligible participant was selected from each household using the ECS eligibility criteria: (1) ≥40 years of age at the time of recruitment, (2) resident in the island for ≥10 years, (3) have no plans to permanently relocate from the island for ≥5 years, (4) able to provide informed consent, (5) English or Spanish language speaking, and (6) have stable contact residential information.

The selection of the household member was performed as follows: (1) The sex, age, and date of birth of each household member 40 years of age and over was recorded on a recruitment form. (2) The person who last celebrated a birthday was selected. In the case of twins, the older sibling was selected. In the case of people with the same birthday, the older of the two was selected.

In the case that (1) an eligible person was not willing to participate in the study, (2) there was no eligible person at the house, or (3) the property was a business or unoccupied house, we moved on to the property at the next interval.

Eligible homes were visited up to 3 times. If no one was at home at the time of the first visit, then at least two subsequent visits were on an evening and a weekend. If no adult was reached after three attempts, the household was removed from the list. Following recruitment, we called each interested participant to schedule an appointment. At least 4 attempts were made to contact people who had been recruited.

## References

[1] World Health Organization Noncommunicable Diseases. https://www.who.int/news-room/fact-sheets/detail/noncommunicable-diseases.

[2] Luciani S., Agurto I., Caixeta R., Hennis A. (2022). Prioritizing noncommunicable diseases in the Americas region in the era of COVID-19. Rev. Panam. Salud Publica.

[3] Razzaghi H., Martin D.N., Quesnel-Crooks S., Hong Y., Gregg E., Andall-Brereton G., Gawryszweski V., Saraiya M. (2019). 10-year trends in noncommunicable disease mortality in the Caribbean region. Rev. Panam. Salud Publica.

[4] Yisahak S.F., Beagley J., Hambleton I.R., Narayan K.V., on behalf of the IDF Diabetes Atlas (2014). Diabetes in North America and The Caribbean: An update. Diabetes Res. Clin. Pract..

[5] Pérez C., Ailshire J.A. (2017). Aging in Puerto Rico: A Comparison of Health Status Among Island Puerto Rican and Mainland U.S. Older Adults. J. Aging Health.

[6] Pabon-Nau L.P., Cohen A., Meigs J.B., Grant R.W. (2010). Hypertension and Diabetes Prevalence Among U.S. Hispanics by Country of Origin: The National Health Interview Survey 2000–2005. J. Gen. Intern. Med..

[7] Layne T.M., Aminawung J.A., Soulos P.R., Nunez-Smith M., Nunez M.A., Jones B.A., Wang K.H., Gross C.P. (2018). Quality of Breast Cancer Care In The US Territories: Insights From Medicare. Health Aff..

[8] U.S. Census Bureau National Caribbean-American Heritage Month: June 2023. Census.gov. https://www.census.gov/newsroom/stories/caribbean-american-heritage-month.html.

[9] NCD Countdown 2030 Collaborators (2018). NCD Countdown 2030: Worldwide trends in non-communicable disease mortality and progress towards Sustainable Development Goal target 3.4. Lancet.

[10] Ouyang F., Cheng X., Zhou W., He J., Xiao S. (2022). Increased Mortality Trends in Patients With Chronic Non-communicable Diseases and Comorbid Hypertension in the United States, 2000–2019. Front. Public Health.

[11] Tamir C. Key Findings about Black Immigrants in the U.S. Pew Research Center. https://www.pewresearch.org/short-reads/2022/01/27/key-findings-about-black-immigrants-in-the-u-s/.

[12] Di Cesare M., Khang Y.-H., Asaria P., Blakely T., Cowan M.J., Farzadfar F., Guerrero R., Ikeda N., Kyobutungi C., Msyamboza K.P. (2013). Inequalities in non-communicable diseases and effective responses. Lancet.

[13] Hospedales C.J., Samuels T.A., Cummings R., Gollop G., Greene E. (2011). Raising the priority of chronic noncommunicable diseases in the Caribbean. Rev. Panam. Salud Publica.

[14] 2011 High Level Meeting on the Prevention and Control of Non-Communicable Diseases. United Nations. https://www.un.org/en/ga/ncdmeeting2011/.

[15] (2022). Global Noncommunicable Disease Programs|Division of Global Health Protection Global Health|CDC. https://www.cdc.gov/globalhealth/healthprotection/ncd/about.html.

[16] (2022). Global Noncommunicable Diseases|Division of Global Health Protection|Global Health|CDC. https://www.cdc.gov/globalhealth/healthprotection/ncd/index.html.

[17] Chronic, Noncommunicable Diseases and Disorders Research Training (D43 D71)—Fogarty International Center @ NIH. Fogarty International Center. https://www.fic.nih.gov:443/Programs/Pages/chronic-lifespan.aspx.

[18] fundsforNGOs (2022). NIH Offers Grants to Implement Research on Noncommunicable Disease. fundsforNGOs. https://www2.fundsforngos.org/latest-funds-for-ngos/nih-offers-grants-to-implement-research-on-noncommunicable-disease/.

[19] Targeting Non-Communicable Diseases and Injuries in Mongolia. Millennium Challenge Corporation. https://www.mcc.gov/resources/doc/evalbrief-0701114-mng-health.

[20] World Health Organization (2013). Global Action Plan for the Prevention and Control of Noncommunicable Diseases 2013–2020.

[21] Sempos C.T., Bild D.E., Manolio T.A. (1999). Overview of the Jackson Heart Study: A Study of Cardiovascular Diseases in African American Men and Women. Am. J. Med. Sci..

[22] Miller G.J., A Beckles G.L., Maude G.H., Carson D.C., Alexis S.D., Price S.G.L., A Byam N.T. (1989). Ethnicity and Other Characteristics Predictive of Coronary Heart Disease in a Developing Community: Principal Results of the St James Survey, Trinidad. Leuk. Res..

[23] Daviglus M.L., Pirzada A., Talavera G.A. (2014). Cardiovascular Disease Risk Factors in the Hispanic/Latino Population: Lessons From the Hispanic Community Health Study/Study of Latinos (HCHS/SOL). Prog. Cardiovasc. Dis..

[24] Smeeton N.C., Corbin D.O.C., Hennis A.J.M., Hambleton I.R., Rose A.M.C., Fraser H.S., Heuschmann P.U., Wolfe C.D.A. (2010). A Comparison of Outcome for Stroke Patients in Barbados and South London. Int. J. Stroke.

[25] Tucker K.L., Mattei J., Noel S.E., Collado B.M., Mendez J., Nelson J., Griffith J., Ordovas J.M., Falcon L.M. (2010). The Boston Puerto Rican Health Study, a longitudinal cohort study on health disparities in Puerto Rican adults: Challenges and opportunities. BMC Public Health.

[26] Lorenzi J., Batalova J. (2022). Caribbean Immigrants in the United States. migrationpolicy.org. https://www.migrationpolicy.org/article/caribbean-immigrants-united-states.

[27] Tripathy J.P. (2018). Research priorities in non-communicable diseases in developing countries: Time to go beyond prevalence studies. Public Health Action.

[28] United States Virgin Islands Population 2023 (Live). https://worldpopulationreview.com/countries/united-states-virgin-islands-population.

[29] Barbados Population 2023 (Live). https://worldpopulationreview.com/countries/barbados-population.

[30] Trinidad and Tobago Population 2023 (Live). https://worldpopulationreview.com/countries/trinidad-and-tobago-population.

[31] Puerto Rico Population 2023 (Live). https://worldpopulationreview.com/countries/puerto-rico-population.

[32] Dietary Screener Questionnaire in the NHANES 2009–10: Background|EGRP/DCCPS/NCI/NIH. https://epi.grants.cancer.gov/nhanes/dietscreen/.

[33] Barbados Statistical Service (2010). Estimated Resident Population* by Age Group and Sex. Stats.gov.bb. https://stats.gov.bb/census/2010-census-tables/2010-census-tables-table-b/..

[34] Pelaez M., Palloni A., Albala C., Alfonso J.C., Ham-Chande R., Hennis A., Lebrao M.L., Lesn-Diaz E., Pantelides E., Prats O. (2006). SABE—Survey on health, well-being, and aging in Latin America and the Caribbean.

[35] Hennis A., Wu S.-Y., Nemesure B., Honkanen R., Leske M.C., Barbados Eye Studies Group (2007). Awareness of Incident Open-angle Glaucoma in a Population Study: The Barbados Eye Studies. Ophthalmology.

[36] U.S. Census Bureau 2010 Island Areas—U.S. Virgin Islands Dataset. Census.gov. https://www.census.gov/data/datasets/2010/dec/virgin-islands.html.

[37] Holtzman D. (2004). The behavioral risk factor surveillance system. Community-Based Health Research: Issues and Methods.

[38] U.S. Census Bureau Puerto Rico Municipios Population Totals: 2010–2019. Census.gov. https://www.census.gov/data/tables/time-series/demo/popest/2010s-total-puerto-rico-municipios.html.

[39] Central Statistical Office Population Statistics. 2010 Cso.gov.tt. https://cso.gov.tt/subjects/population-and-vital-statistics/population/.

[40] Miljkovic I., Bodnar L.M., A Cauley J., Bunker C.H., Patrick A.L., Wheeler V.W., Kuller L.H., Zmuda J.M. (2011). Low prevalence of vitamin D deficiency in elderly Afro-Caribbean men. Ethn. Dis..

[41] Continuous Sample Survey of Population. http://laborsta.ilo.org/applv8/data/SSM3/E/TT.html.

[42] PanAm STEPS Survey Final Report: Trinidad and Tobago. http://www.health.gov.tt/news/newsitem.aspx?id=394.

